# Isolated complete avulsion of the gallbladder (near traumatic cholecystectomy): a case report and review of the literature

**DOI:** 10.1186/1752-1947-5-392

**Published:** 2011-08-18

**Authors:** Theodoros E Pavlidis, Miltiadis A Lalountas, Kyriakos Psarras, Nikolaos G Symeonidis, Anastasios Tsitlakidis, Efstathios T Pavlidis, Konstantinos Ballas, Nikolaos Flaris, Georgios N Marakis, Athanassios K Sakantamis

**Affiliations:** 1 Second Surgical Propedeutical Department, Medical School, Aristotle University of Thessaloniki, Hippocration Hospital, Konstantinoupoleos 49, 546 42 Thessaloniki, Greece

## Abstract

**Introduction:**

Injury of the gallbladder after blunt abdominal trauma is an unusual finding; the reported incidence is less than 2%. Three groups of injuries are described: simple contusion, laceration, and avulsion, the last of which can be partial, complete, or total traumatic cholecystectomy.

**Case presentation:**

A case of isolated complete avulsion of the gallbladder (near traumatic cholecystectomy) from its hepatic bed in a 46-year-old Caucasian man without any other sign of injury is presented. The avulsion was due to blunt abdominal trauma after a car accident. The rarity of this injury and the stable condition of our patient at the initial presentation warrant a description. The diagnosis was made incidentally after a computed tomography scan, and our patient was treated successfully with ligation of the cystic duct and artery, removal of the gallbladder, coagulation of the bleeding points, and placement of a drain.

**Conclusions:**

Early diagnosis of such injuries is quite difficult because abdominal signs are poor, non-specific, or even absent. Therefore, a computed tomography scan should be performed when the mechanism of injury is indicated.

## Introduction

The first specimen of a lacerated gallbladder from a blunt trauma was found in Guy's Museum in London and dates from 1388 [1]. The first known case of someone surviving a gallbladder traumatic rupture was in 1898 [1]. Penn [2] reported the incidence of gallbladder trauma to be 1.9% in a collected review of 5670 cases of blunt and penetrating trauma. Complete detachment of the gallbladder from its hepatic bed, one of the rarest consequences of blunt abdominal trauma, is rarer than gallbladder contusion, perforation, and partial contusion. The few reports in the literature are not clearly enumerated [3-9], because of a lack of appropriate description before the advanced classification of Losanoff and Kjossev [4].

The gallbladder is a well-protected organ, being partially embedded in the relatively massive liver substance, cushioned on the surrounding omentum and intestines, and covered by the bony cartilaginous rib cage. As a result, gallbladder trauma due to a blunt injury is rare and usually is associated with additional external or visceral injuries [2,5,6,8]. Isolated complete avulsion of the gallbladder after non-penetrating abdominal trauma in a stable patient without any other sign of injury is even rarer and is prone to delayed diagnosis and treatment [5,9]. A computed tomography (CT) scan should be performed when the mechanism of injury is indicated, and an early explorative laparotomy is recommended to reduce the high morbidity associated with this condition [10-12].

## Case presentation

A 46-year-old Caucasian man was involved in a car accident. He was a pedestrian when a car hit him. He fell down on the road and one of the car's rear wheels rolled over his lower chest. Two hours later, he presented in our emergency department. On admission, he was complaining of bilateral hypochondrial pain radiating to his right shoulder; he was hemodynamically stable after repeated blood tests and had a blood pressure of 130/100 mm Hg and a pulse rate of 90 beats per minute. An examination revealed no chest or abdominal wall contusions. A chest X-ray was normal and there were no rib fractures. The results of an ultrasound (US) examination of the abdomen were normal, but the gallbladder could not be visualized.

**Figure 1:**
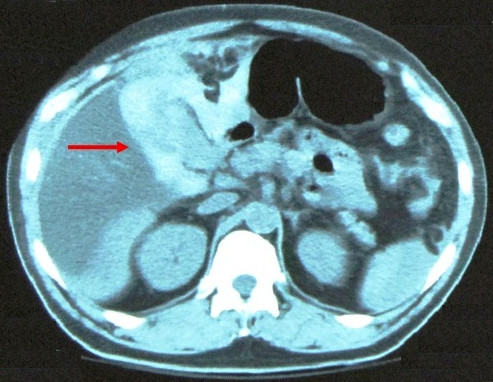
**Computed tomography (CT) scan reveals pericholecystic fluid (arrow) and indicates the potential for some kind of injury of the gallbladder**.

. The results of all laboratory tests were normal except for a leucocytosis level of 12.2 × 10^3^/mm^3^. Because of the suspicion of possible intra-abdominal injury due to the severe mechanism of the accident, a CT scan was performed. The scan revealed pericholecystic fluid and the possibility of an avulsed gallbladder (Figure 1). Magnetic resonance imaging (MRI) would have been another option, but our patient had a contraindication because of the presence of a pacemaker. An exploratory laparotomy was performed five hours after admission, although our patient remained hemodynamically stable.

**Figure 2:**
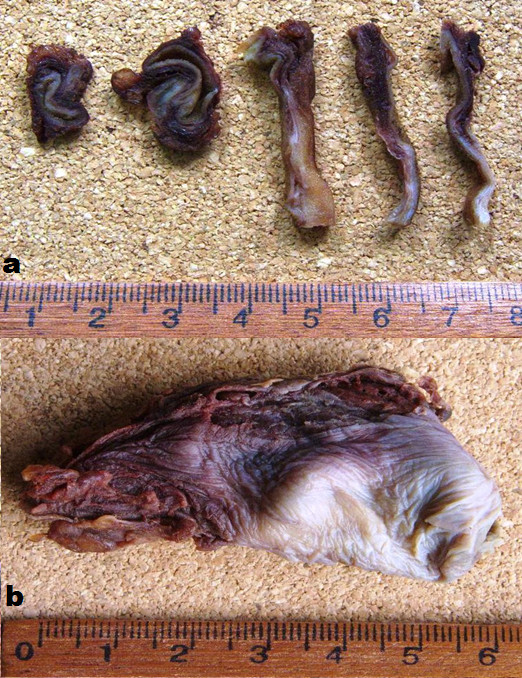
**Photographs of fixed gallbladder prepared with formaldehyde**. (a) Successive sections of the gallbladder show traumatic hemorrhagic filtering. (b) Inverted gallbladder with the same findings.

During the laparotomy, a moderate amount of fresh blood was identified in the right subhepatic space. The gallbladder was lying freely avulsed, detached from its liver bed, but there was no extrahepatic bile duct injury. The gallbladder's attachments to the cystic duct and the cystic artery were intact and both of these structures were subsequently ligated. The removed gallbladder contained no stone. The abdomen had no other pathology and was washed, drained, and closed in layers. The postoperative course was uneventful, and our patient was discharged on the fifth postoperative day. A pathology report confirmed gallbladder injury with hemorrhage and chronic cholecystitis (Figure 2a, b).

## Discussion

**Figure 3:**
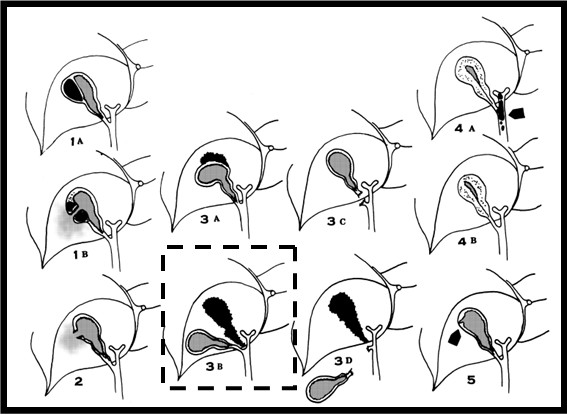
**Schematic drawing of all known types of gallbladder injury according to the classification by Losanoff and Kjossev **[4]. Our case is highlighted.

Blunt gallbladder injuries are classified as contusion, perforation, or avulsion [4-6,8]. Contusion, defined as an intramural hematoma, is most often diagnosed at the time of laparotomy and is probably underreported. Perforation, also known as "rupture" or "laceration", is the most commonly reported injury. Avulsion has three subtypes: partial avulsion, in which the gallbladder is partially detached from the liver bed; complete avulsion, in which the gallbladder is completely detached from the liver bed but the cystic duct and artery are intact; and total avulsion, in which the gallbladder lies free in the abdomen, torn from all attachments. To the best of our knowledge, only eight cases of total avulsion (also called "traumatic cholecystectomy") have been reported. Traumatic cholecystitis is caused by a cystic duct obstruction by blood clots from a liver or gallbladder injury. Losanoff and Kjossev [4] describe a more detailed classification of blunt gallbladder injuries; according to their classification, our patient belongs to type 3B (isolated complete avulsion of the gallbladder or near traumatic cholecystectomy; Figure 3 and Table 1[4]).

**Table 1:** Types of gallbladder injury according to the classification by Losanoff and Kjossev [4] (Figure 3)

Type	Injury of the gallbladder
1A	Contusion with intramural hematoma
1B	Contusion with perforation
2	Rupture
3A	Avulsion with partial detachment
3B	Avulsion with complete detachment from the liver but with attachment to the structures of the hepatoduodenal ligament (so-called "near traumatic cholecystectomy")
3C	Torn only from the hepatoduodenal ligament
3D	Completely torn from all attachments (so-called "traumatic cholecystectomy")
4A	Traumatic cholecystitis, secondary to hemobilia
4B	Acute acalculus cholecystitis
5	Mucosal tear with leakage of bile

Earlier reports indicate that the most common etiologic factors in blunt trauma were falls, kicks, or blows. At present, motor vehicle crashes are the predominant cause of blunt gallbladder trauma [2,4,5,8]. Factors predisposing people to blunt gallbladder injuries are a thin-walled normal gallbladder, a distended gallbladder, and alcohol ingestion, the last of which increases the tone of sphincter of Oddi and the biliary tract pressure. Our patient had a history of chronic alcohol consumption.

Associated intra-abdominal injuries are common in patients with a blunt gallbladder injury, averaging 2.7 to 3.3 associated injuries per patient. Liver injury is especially likely; the reported incidence is 83% to 91%. Duodenum and spleen injuries occur in up to 54% of patients with a blunt gallbladder injury [4,8]. Our patient had no other injuries. We used ultrasonography initially because of its low cost and the ability to perform the test at the bedside in the emergency department.

Non-visualization of the gallbladder at ultrasonography should raise the suspicion of a traumatic gallbladder avulsion or rupture [7-12]. CT findings of gallbladder injury are largely non-specific. Pericholecystic fluid is most common but is least specific. Other signs of gallbladder injury are an ill-defined contour of the gallbladder wall, a mass effect on the duodenum, high-attenuation intraluminal material (blood), a thickened gallbladder wall, and a collapsed gallbladder in a fasting patient. Also, major liver injury often dominates the CT picture and overshadows subtle abnormalities of the gallbladder. It is not surprising that unsuspected gallbladder injury is often discovered during a laparotomy for coexisting intra-abdominal injuries. Gallbladder injuries, though infrequent, can be difficult to diagnose. CT is the most reliable technique to diagnose a gallbladder injury. However, benign entities can mimic gallbladder injury. Delayed images through the gallbladder can be useful in differentiating between a true gallbladder injury and a relatively benign process [13]. In our case, the possibility of an avulsed gallbladder was revealed from an abdominal CT scan, which was performed because of the severe mechanism of the accident. An abdominal CT scan, rather than US or MRI, is considered the "gold standard" method to diagnose this kind of injury [10-13]. In such cases, we recommend that a CT scan be performed, even in the absence of other signs of injury in a hemodynamically stable patient.

The choice of treatment depends on the severity of the gallbladder injury and the general condition of the patient. Patients with mild injuries such as contusion or isolated partial avulsion may be observed, although late necrosis and perforation have been reported [9,14,15]. Severe injuries generally require a cholecystectomy [16]. When the patient is hemodynamically stable, a diagnostic laparoscopy could play a role. Laparoscopic surgical techniques may be safely used when the likelihood of associated injuries is low and definitive treatment can be rendered without increasing patient morbidity and mortality [17,18].

## Conclusions

Early diagnosis of gallbladder injuries, such as near traumatic cholecystectomy, is quite difficult because abdominal signs are poor, non-specific, or even absent. Therefore, a CT scan should be performed when the mechanism of injury is indicated. Such injuries have a good prognosis if they are diagnosed early and there is no serious associated trauma. Trauma surgeons should always be aware of the existence of these injuries.

## Abbreviations

CT: computed tomography; MRI: magnetic resonance imaging.

## Consent

Written informed consent was obtained from the patient for publication of this case report and any accompanying images. A copy of the written consent is available for review by the Editor-in-Chief of this journal.

## Competing interests

The authors declare that they have no competing interests.

## Authors' contributions

TEP performed the procedure. MAL obtained the patient's written informed consent to publish the report, conducted the follow-up examinations, analyzed and interpreted the patient data, and wrote part of the manuscript. KP, NGS, AT, and ETP edited and wrote part of the manuscript. KB and GNM were major contributors to the review and editing of the manuscript. NF was the main pathologist and revised the manuscript. AKS made the strategic plan and gave the final approval. All authors read and approved the final manuscript.
